# The siRNA cocktail targeting VEGF and HER2 inhibition on the proliferation and induced apoptosis of gastric cancer cell

**DOI:** 10.1007/s11010-013-1850-0

**Published:** 2013-10-26

**Authors:** Kun Liu, Honglin Chen, Qingsheng You, Hai Shi, Zhiwei Wang

**Affiliations:** 1 Department of Cardiothoracic Surgery, The Affiliated Hospital of Nantong University, 20 Xisi Road, Nantong, 226001 Jiangsu People’s Republic of China; 2 Nursing School of Nantong University, 19 Qixiu Road, Nantong, 226001 Jiangsu People’s Republic of China

**Keywords:** siRNA, siRNA cocktail, VEGF, HER2, Gastric cancer, SGC-7901

## Abstract

The aim of this study was to investigate the inhibitory effect of a siRNA cocktail targeting Vascular endothelial growth factor (VEGF) and Human epidermal growth factor receptor 2 (HER2) on cell proliferation, induced apoptosis and the expression of VEGF and HER2 in human gastric carcinoma cell. The silencing rate of pre-designed siRNAs that targeted VEGF and HER2 was detected by Real-time Quantitative PCR (RT-QPCR) analysis. Furthermore, the best silencing siRNA that targeted VEGF and HER2 was prepared as a cocktail to co-knockdown VEGF and HER2 expression at both mRNA and protein levels which were detected by RT-QPCR and Western blot analysis. Cell proliferation inhibition rates were determined by CCK8 assay. The effect of siRNA cocktail on cell apoptosis was determined by flow cytometry. The migration inhibition of siRNA cocktail was analyzed by wound-healing assay. The ability of VEGF to induce endothelial cells to proliferate was examined in HUVECs by the method of tube formation assay. The pre-designed siRNAs could inhibit VEGF and HER2 mRNA level. siRNA cocktail, and co-downregulation of VEGF and HER2 result in significant inhibition of gastric cancer growth and migration in vitro. The inhibition of VEGF and HER2 expressions can induce apoptosis of SGC-7901 cells.

## Introduction

Gastric cancer is one of the most common types of cancer and the second most common cause of cancer-related mortality worldwide [1, 2]. At present, conventional therapies for gastric cancer include resection, chemotherapy, and radiotherapy, but these measures are non-curative for those patients who are diagnosed with advanced gastric cancer. As a result, new therapeutic methods are needed urgently for more effective treatment of this aggressive malignancy. Biological therapy based on the molecular-targeted therapy is an emerging technology for gastric cancer to improve the quality of life and survival of patients.

RNA interference (RNAi) is a post-transcriptional process triggered by double-stranded RNA which leads to gene silencing in a sequence-specific manner through degradation of the corresponding mRNA.

Human epidermal growth factor receptor 2 (HER2), also known as ErbB-2, is a protein that in humans is encoded by the ERBB2 gene. HER2 is a member of the epidermal growth factor receptor (EGFR/ErbB) family. Its activation leads to a cascade of events promoting rapid cell growth, differentiation, survival, and migration [3]. Overexpression of HER2 has been found to induce tumorigenesis and to be involved in the pathogenesis of gastric cancer [4]. Amplification and overexpression of HER2 play an important role in disease initiation, progression, and metastasis, and have been associated with a worse prognosis in patients with gastric cancers [5].

Vascular endothelial growth factor (VEGF) is a signal protein produced by cells that stimulates vasculogenesis and angiogenesis. It is the key mediator of angiogenesis in cancer, in which it is up-regulated by oncogene expression, a variety of growth factors, and also hypoxia [6]. And VEGF is correlated with gastric cancers, especially in the patients with advanced gastric cancer which significantly lowers survival rates. VEGF can serve as a pertinent prognostic indicator both in early and advanced gastric cancer [7]. Bevacizumab, a monoclonal antibody targeting VEGF-A, was used to inhibit advanced gastric cancer combined with chemotherapy [8].

In the present study, we intended to use siRNA cocktail therapy which targets VEGF and HER2 gene for the treatment of gastric cancer. Pre-designed VEGF and HER2 siRNAs were screened in gastric cancer cells, and the best siRNA targets were used as cocktail to inhibit the growth and induced apoptosis of SGC-7901 gastric cancer cell.

## Materials and methods

### Cell culture

Gastric cancer SGC-7901 and HUVEC cell lines were purchased from the Institute of Cell Biology, Chinese Academy of Sciences. The cells were cultured in Dulbecco modified Eagle medium (DMEM) (Gibco, USA) supplemented with 10 % fetal bovine serum (FBS) (Gibco, USA), 100 U/ml penicillin, and 100 μg/ml streptomycin (Invitrogen, USA) at 37 °C in a humidified incubator with 5 % CO_2_.

### siRNA sequences and transfection in vitro

The sequence of VEGF and HER2 was obtained from GenBank (Assession No. NM_001171623 and NM_001005862). According to an optimization principle of siRNA design methods [9], we designed 4 sequence-specific siRNAs targeting either VEGF or HER2 in length of 19 nt with dTdT 3′overhang. Meanwhile, negative control siRNA (NC-siR) that has no homology with human genome was designed as negative control. The sequences used for the experiments are shown in Table 1. All chemically synthesized siRNAs were obtained from Biomics Biotech (China).

**Table 1:** Sequences of pre-designed siRNAs targeting either VEGF or HER2

Name	Sequences (5′–3′)
VEGF_siR1	Sense GCUUCCUACAGCACAACAAdTdT
	Antisense UUGUUGUGCUGUAGGAAGCdTdT
VEGF_siR2	Sense AGAUCGAGUACAUCUUCAAdTdT
	Antisense UUGAAGAUGUACUCGAUCUdTdT
VEGF_siR3	Sense UGAAGUUCAUGGAUGUCUAdTdT
	Antisense UAGACAUCCAUGAACUUCAdTdT
VEGF_siR4	Sense GCCUUGCCUUGCUGCUCUAdTdT
	Antisense UAGAGCAGCAAGGCAAGGCdTdT
HER2_siR1	Sense CCUGUUCUCCGAUGUGUAAdTdT
	Antisense UUACACAUCGGAGAACAGGdTdT
HER2_siR2	Sense GCUUUGUGGUCAUCCAGAAdTdT
	Antisense UUCUGGAUGACCACAAAGCdTdT
HER2_siR3	Sense GGUGUGAGAAGUGCAGCAAdTdT
	Antisense UUGCUGCACUUCUCACACCdTdT
HER2_siR4	Sense GUGUGGACCUGGAUGACAAdTdT
	Antisense UUGUCAUCCAGGUCCACACdTdT
NC_siR	Sense UUCUCCGAACGUGUCACGUdTdT
	Antisense ACGUGACACGUUCGGAGAAdTdT

All the above siRNAs were mixed into Lipofectamine^™^ 2000 (Invitrogen, USA) and transfected according to the manufacturer’s instruction. After 6 h at 37 °C, the DMEM was replaced with complete growth medium (DMEM with 10 % FBS). The cells without siRNA transfection were used as untreated control.

### RT-QPCR analysis

The mRNAs were isolated from SGC-7901 cells using TurboCapture 96 mRNA Kit (QIAGEN, USA) 48 h post-transfection, and then were submitted to a 25 μl PCR reaction in the presence of 12.5 μl of 2× Master Mix, 1 μl of each forward and reverse primers mix (10 μM each), 0.5 μl of 50× SYBR Green I and 4 μl mRNA as template. The PCR mixtures were first subjected to reverse transcription for 30 min at 42 °C and initially denatured for 5 min at 95 °C, and then to 45 cycles of amplification with the following cycling condition: 20 s at 95 °C, 30 s at 58 °C, and 30 s at 72 °C. The primer pairs for each gene were designed with Primer Premier 5.0 software; glyceraldehyde-3-phosphate dehydrogenase served as an internal control for PCR. Sequences of all the primers (Biomics Biotech, China) are shown in Table 2.

**Table 2:** Sequences of the primers for RT-QPCR

Name	Sequence (5′–3′)
VEGF	Forward: CTGTACCTCCACCATGCCAAGT
	Reverse: CTTCGCTGGTAGACATCCATGA
HER2	Forward: TCCGTTTCCTGCAGCAGTCTCCGCA
	Reverse: AGAGAGCCAGCCCTCTGACGTCCAT
GADPH	Forward: GGTCTCCTCTGACTTCAACA
	Reverse: AGCCAAATTCGTTGTCATAC

### Western blot analysis

The protein expression levels of SGC-7901 cells were measured by Western Blot analysis. Cells were plated at a concentration of 1 × 10^6^ cells per well in 6-well plates, and grown for 24 h until they reached 70–80 % confluence. The cells were divided into five groups with different treatments at final concentration of 20 nM: VEGF_siR4, HER2_siR3, siRNA cocktail (VEGF_siR4 mixed with HER2_siR3 at equal concentration), NC_siR, and without treated as untreated group. Cells were harvested at 48 h after transfection and lysed in ice-cold cell RIPA buffer (Beyotime, China). The amount of total cell proteins was determined by BCA kit (Beyotime, China). 20 μg of proteins were separated on SDS-PAGE and electroblotted onto PVDF membranes (Millipore, USA). Then the membranes were blocked with 5 % skim milk in TBST (10 mM Tris–HCl, 150 mM NaCl, 0.25 % Tween 20, pH 7.5) at room temperature for 2 h, following incubated overnight with primary antibody of polyclonal rabbit-anti-human VEGF (Gene Biotech, USA; 1:200 dilution) and mouse-anti-hunan HER2 (Santa Cruz Biotech, USA; 1:200 dilution), with mouse-anti-human β-actin (Santa Cruz Biotech, USA; 1:200 dilution) as control. After washing with TBST, the membranes were incubated with secondary antibody (Santa Cruz Biotech, USA; goat anti-rabbit IgG-HRP with 1:1,000 dilution for VEGF; goat anti-mouse IgG-HRP with 1:2,000 dilution for HER2 and β-actin) for 2 h at room temperature. After three washes with TBST, proteins were visualized by chemiluminescence. The relative amount of proteins on the blots was determined by Image J software.

### Cell proliferation assay

SGC-7901 cells proliferation was measured by Cell Counting Kit-8 detection kit (Dojindo, Japan). Cells were seeded at a concentration of 5 × 10^3^ cells per well in 96-well plates. All experiments were conducted in triplicate. After grown for 24 h resulted in about 70–80 % confluence, cells were performed with different treatments as described above. At 24, 48, 72 and 96 h after transfection, CCK-8 solution was applied at 10 μl per well and followed by 2-h incubation at 37 °C. Absorbance values of all wells were then determined at 450 nm in Microplate Reader (Bio-Rad, USA).

### Wound-healing assay

Cells migration was measured by wound-healing assay. SGC-7901 cells were seeded and transfected with siRNAs as described above in 12-well plates at the density of 3 × 10^5^ cells per well. After 48 h, wound was made through confluent monolayer cells with a pipette tip and cells were washed with PBS, DMEM medium without adding FBS. Photographs of cells were taken at 0, 24, 48, and 72 h to monitor cell movements.

### FCM analysis

Annexin-V/propidium iodide (PI) double staining assay was performed by using the Annexin V-FITC apoptosis detection kit (Sigma-aldrich, USA) as described by the manufacture’s instruction. Briefly, 1 × 10^6^ cells per well after 48-h transfection with various treatments described above were harvested and washed with PBS in 6-well plates. The cells were washed with PBS twice and resuspended in 1× Binding buffer, followed by incubated with Annexin V-FITC conjugate and PI for 15 min to protect from light at room temperature. Cells were analyzed by flow cytometry (FCM) analysis using BD CELLQuest software (BD Biosciences, USA).

### Tube formation assay

The ability of endothelial cells to sprout new blood vessels stimulated by pro-angiogenic factors released from the gastric cancer cell was examined in HUVECs angiogenesis in vitro model [10]. Briefly, 6 × 10^4^ HUVECs were collected, resuspended in a conditioned medium, which was the supernatant of SGC-7901 with siRNAs post-transfected for 48 h, seeded in 48-well plates that were coated with 100 μl of gelled matrigel (BD Biosciences, USA), and cultured in a humidified 37 °C/5 % CO_2_ incubator. After incubation for 6 or 12 h, numbers of branching points were counted. The data were obtained from triplicate wells under each experimental condition at each time point.

### Statistical analysis

All experiments were performed independently three times, the results a were shown as mean values ± standard deviation (SD), and statistical analyses were performed using SPSS17.0 software. The statistical differences were calculated using a standard one-way ANOVA and two-tailed unpaired Student’s t test. *P* < 0.05 was considered as statistically significant. In all graphs, *, # indicates significant difference.

## Results

### Inhibition effects of pre-designed siRNAs that target VEGF and HER2

**Fig. 1:**
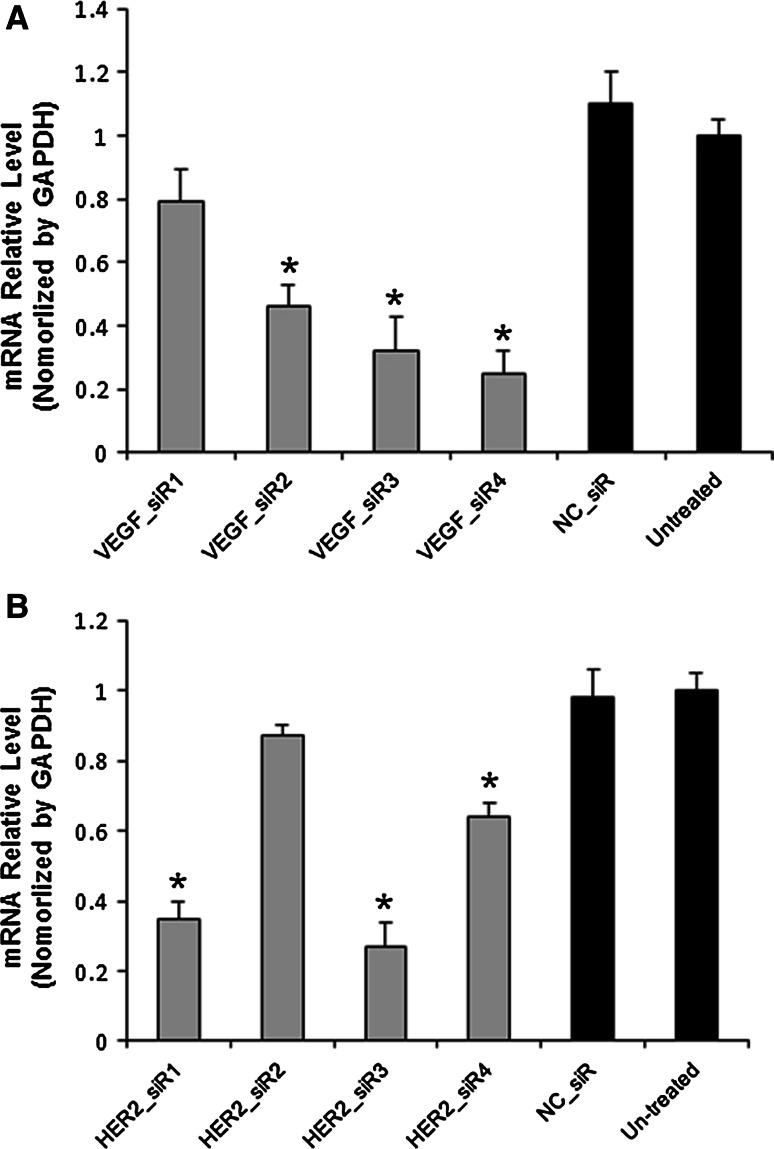
The mRNA relative level of VEGF (**a**) and HER2 (**b**) with pre-designed siRNAs treatments in SGC-7901 cells by RT-QPCR. Values were given as mean ± SD of three separate experiments with triplicate wells per condition. ^*^ *P* < 0.01 compared with untreated and NC_siR

The mRNA levels were determined by RT-QPCR after treatments with SGC-7901 cells for 48 h. As shown in Fig. 1a, VEGF siRNAs inhibited the VEGF expression at the mRNA level up to 75 % in comparison to the untreated ones. And the silencing effects of HER2 siRNAs were observed at mRNA level up to 73 % (Fig. 1b).

### Inhibition effects of siRNA cocktail targeting VEGF and HER2 on mRNA and protein level

**Fig. 2:**
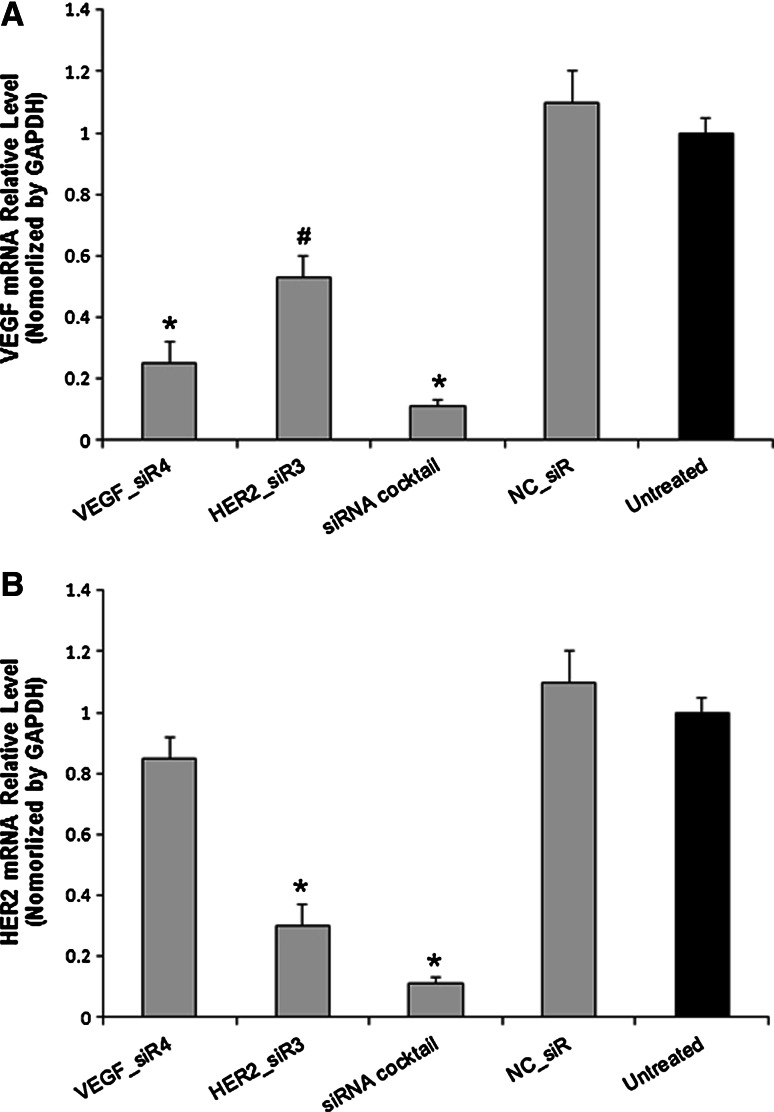
The mRNA relative level of VEGF (**a**) and HER2 (**b**) with different treatments in SGC-7901 cells by RT-QPCR. Values were given as mean ± SD of three separate experiments with triplicate wells per condition. ^*^ *P* < 0.01 compared with untreated, ^#^ *P* < 0.05 compared with untreated

**Fig. 3:**
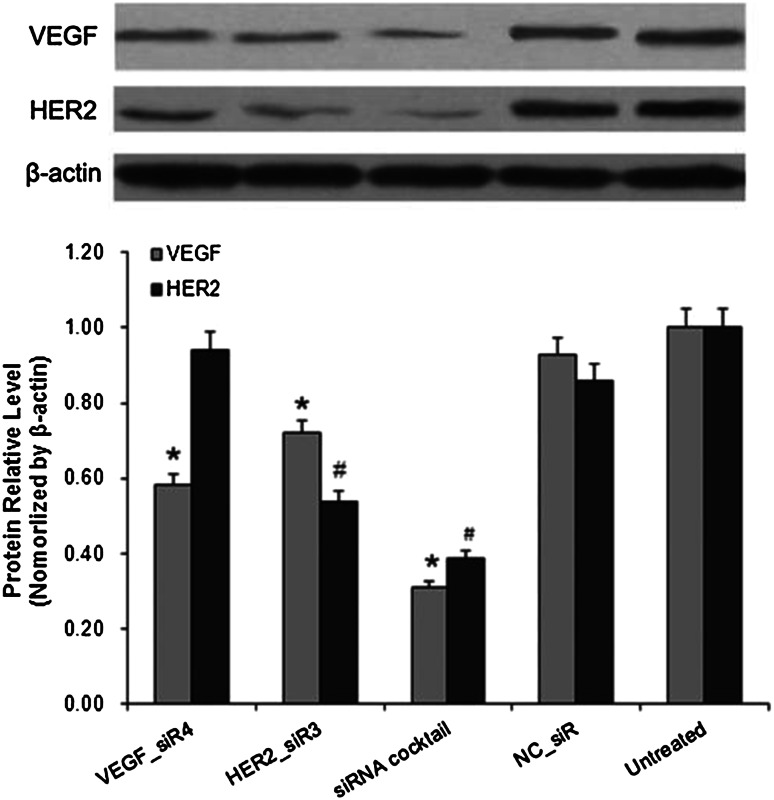
The protein relative level of VEGF and HER2 with different treatments in SGC-7901 cells by Western blot. Values were given as mean ± SD of three separate experiments with triplicate wells per condition. ^*^ *P* < 0.05 VEGF protein relative level compared with untreated and NC_siR, ^#^ *P* < 0.05 HER2 protein relative level compared with untreated and NC_siR

After treatments of siRNA cocktail and controls into SGC-7901 cells for 48 h, the mRNA and protein level of VEGF and HER2 was determined by RT-QPCR and Western blot. As shown in Figs. 2 and 3, siRNA cocktail inhibited the VEGF and HER2 expressions at the mRNA and protein level, obviously in comparison to the untreated ones. Meanwhile, HER2 was silenced by HER2_siR3, but VEGF was also inhibited by it at mRNA level up to 47 % (*P* < 0.05), and the detection of protein expression was confirmed by knockdown (Fig. 3).

### Inhibition of cell proliferation by siRNA cocktail targeting VEGF and HER2

**Fig. 4:**
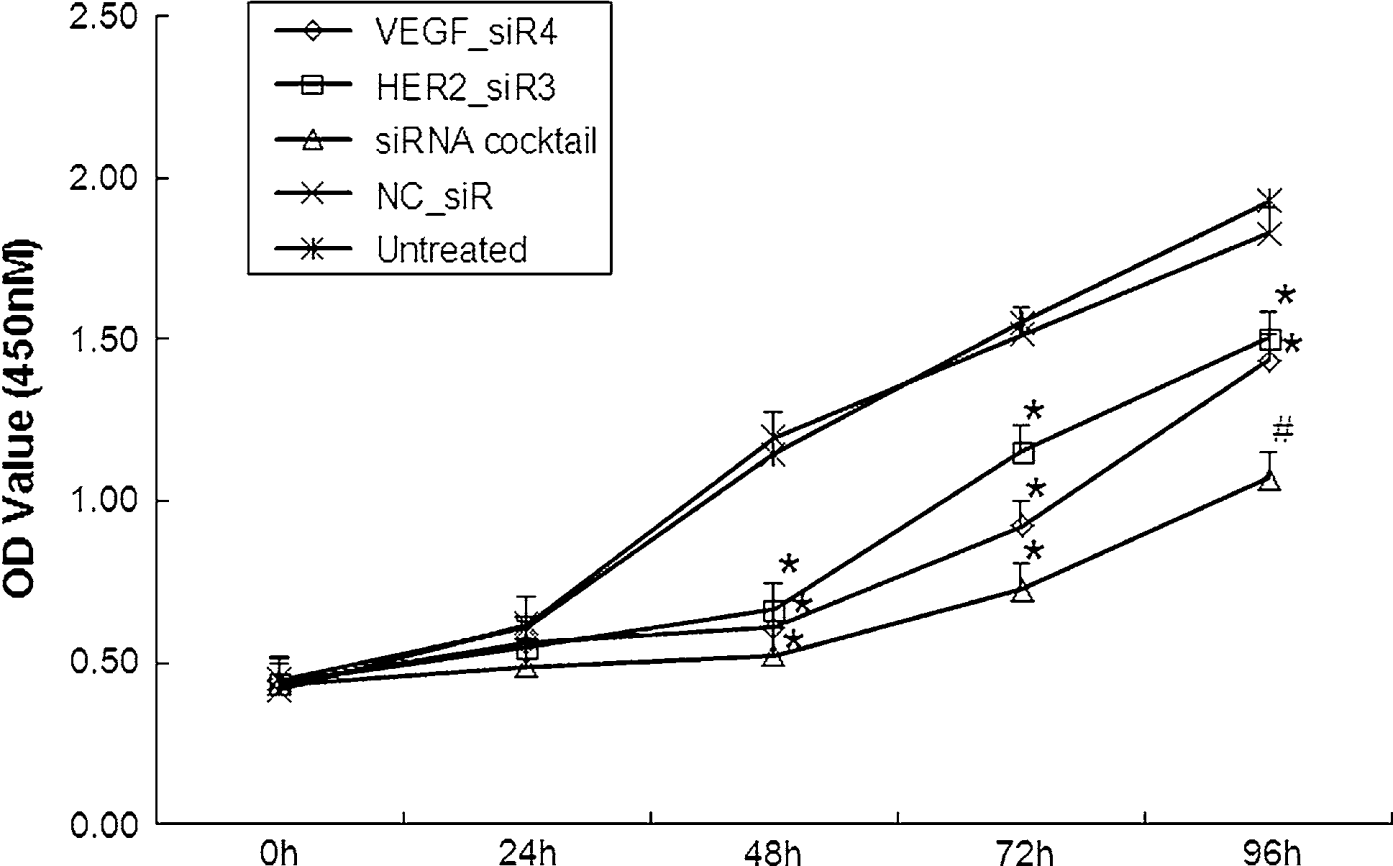
Cell proliferation was analyzed by CCK8 assay. Growth curve of SGC-7901 cells was shown for each treatment at 0, 24, 48, 72, and 96 h. ^*^ *P* < 0.01 compared with untreated, ^#^ *P* < 0.05 compared with untreated

The cell proliferation inhibition effects of siRNA cocktail in SGC-7901 cells were detected by CCK8 assay. The absorbance values of the SGC-7901 cells at 48, 72 and 96 h post-transfection with siRNA cocktail and either VEGF_siR4 or HER2_siR3 were significantly lower than those of the untreated cells. There was no significant difference between the growth of cells treated with VEGF_siR4 and that of HER2_siR3. The cell proliferation inhibition rate treated with siRNA cocktail showed a significant decrease in cell proliferation compared with the cells treated with either VEGF_siR4 or HER2_siR3 at 48, 72 and 96 h (Fig. 4).

### Decrease of the cell migration ability by siRNA cocktail targeting VEGF and HER2

**Fig. 5:**
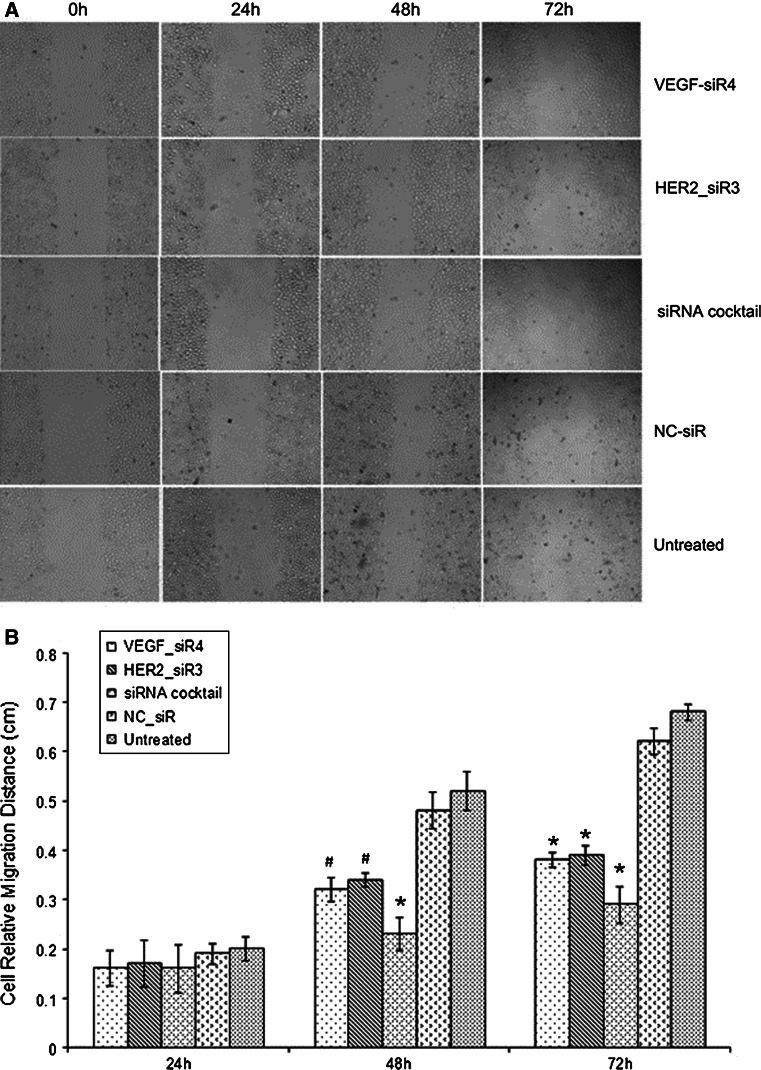
Cell migration inhibition effects of targeting VEGF and/or HER2 on SGC-7901. **a** The cells with different treatments at 0, 24, 48, and 72 h (100×). **b** Cell relative migration distances in different time point treatments were presented as mean ± SD. ^*^ *p* < 0.01, ^#^ *p* < 0.05 compared with untreated and NC_siR

Wound-healing assay was used to evaluate the migration ability of SGC-7901 cells after different treatments. As illustrated in Fig. 5a, the scratch caused in groups of untreated and NC_siR nearly closed completely after 72 h, but the cells in treatment with siRNA cocktail and VEGF_siR4 or HER2_siR3 were not able to move toward the center of the wound. As shown in Fig. 5b, siRNA cocktail and VEGF_siR4 or HER2_siR3 all exhibited a decrease in wound healing ability compared to the untreated and NC_siR-treated cells (^*^ *P* < 0.01).

### Cell apoptosis induced by siRNA cocktail that targeting VEGF and HER2

**Fig. 6:**
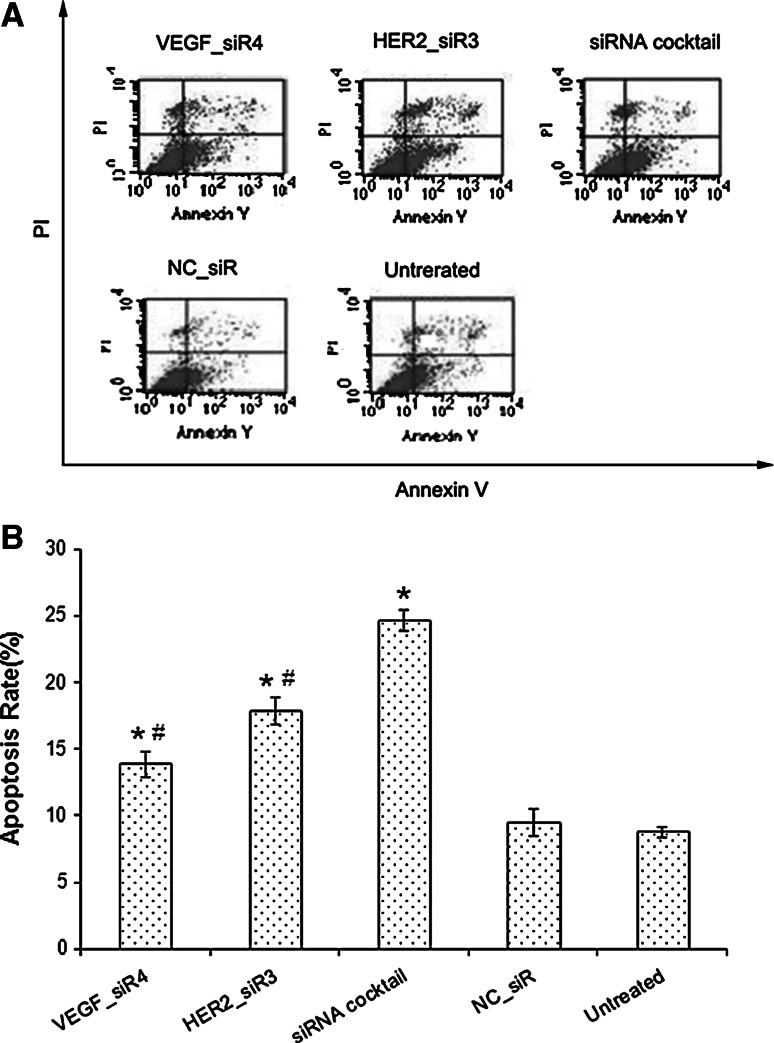
Cell apoptosis was detected by Annexin V-FITC/PI double staining and FCM analysis. **a** Apoptosis rates were measured by FCM analysis after AnnexinV/PI staining. **b** Percentage of dual-positive (Annexin V and PI were positive) cells from three independent experiments was quantified and presented as mean ± SD. ^*^ *p* < 0.01 compared with untreated and NC_siR, ^#^ *p* < 0.05 compared with siRNA cocktail

Annexin V-FITC/PI double staining and FCM analysis were performed to evaluate the ability of siRNA cocktail, VEGF_siR4, or HER2_siR3 on inducing SGC-7901 cell apoptosis. As Fig. 6 shows, treatment with siRNA cocktail resulted in a significant increase of apoptosis compared with that of untreated cells (*p* < 0.05).

### Influence of siRNA cocktail targeting VEGF and HER2 on tube formation

**Fig. 7:**
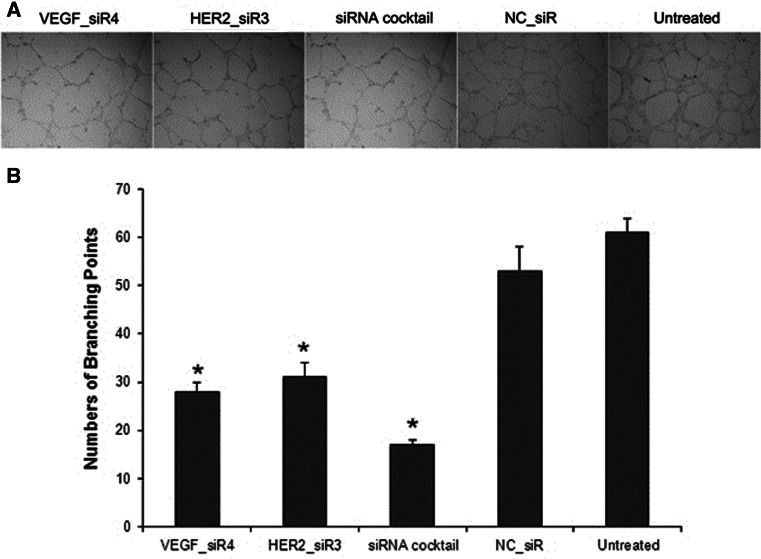
The tube formation inhibition of siRNAs was detected in HUVECs angiogenesis model. **a** Representative photographs of each treatments were shown (100×). **b** The total numbers of branching points were decreased treated by siRNA cocktail, VEGF_siR4, and HER_siR3 compared with the untreated and NC_siR (^*^ *P* < 0.01)

A HUVECs angiogenesis model was employed to evaluate the tube formation of HUVECs stimulated by the conditioned medium derived from SGC-7901 cells transfected with siRNA cocktail, VEGF_siR4, HER2_siR3 and NC_siR. As illustrated in Fig. 7, HUVECs which were treated with siRNA cocktail, VEGF_siR4, HER2_siR3 were inhibited to form extensive and enclosed tube networks as compared with the untreated ones.

## Discussion

In the present study, we found that siRNA cocktail silencing VEGF and HER2 together could inhibit the proliferation and migration of gastric cancer cell very well. On the other hand, the cocktail could also promote apoptosis of gastric cancer cell.

HER2 was overexpressed in several cancers, such as breast cancers [11], colon [12], bladder [13], ovarian [14], endometrial [15], lung [16], uterine cervix [17], head and neck [18], esophageal [19] etc. HER2 proteins have been shown to form clusters in cell membranes that may play a role in tumorigenesis [20, 21].

In recent years, HER2 was found overexpressed in gastric cancer; it could be a new prognostic factor and a novel therapeutic target [22, 23]. Some reports have shown that siRNA-mediated knock-down of HER2 expression resulted in decreased proliferation and induced apoptosis in breast cancer cell SKBr3 and the ovarian cancer cell [24]. In this study, we also demonstrated that the siRNA targeting HER2 could inhibit proliferation and induce apoptosis in SGC-7901 gastric cancer cells.

Generally, tumors do need to induce a new vascular supply if they are to grow [25]. VEGF is widely expressed by nearly all malignant tumors, and it acts on vascular endothelial cells to induce vascular permeability and, in the longer term, reprograms gene expression, leading to endothelial cell proliferation and migration in vitro, and generation of new blood vessels in vivo. Moreover, a variety of anti-tumor drugs that target VEGF-A or its receptors had been demonstrated effectively [26, 27]. A humanized antibody drug-bevacizumab against VEGF-A prolongs the life of patients with advanced colon cancer by, on average, 4–5 months, and then only when combined with triple chemotherapy [28]. In fact, a recent editorial has shown its limited effectiveness, serious side effects, and high cost [29].

RNAi is a new approach for therapeutic treatment of cancer, infectious diseases, and other diseases associated with specific gene disorders, with more than 30 clinical trials of RNAi-based drugs since the discovery of the mechanism [30]. Furthermore, the siRNA conjugating with the delivery system showed safety on the clinical trial [31]. The Anti-VEGF siRNA had already been reported by many researchers for the cancer treatment [24, 32, 33]. And, VEGF is also overexpressed in advanced gastric cancer [7]. In the study, we found that siRNA targeting VEGF could inhibit gastric cancer cell proliferation and anti-angiogenesis better.

Using the siRNA cocktail targeting VEGF and HER-2 to inhibit the proliferation and induce apoptosis of gastric cancer cell is the first report by our study. Gastric cancer cell SGC-7901 with overexpression of VEGF and HER2 was used as in vitro cancer model; the best siRNAs for knockdown of the VEGF and HER2 were screened by RT-QPCR. Furthermore, we prepared the siRNA cocktail of best siRNAs, analyzed the cell treated with siRNA cocktail and controls (including single siRNA targeting VEGF or HER2 and negative control siRNA which has no homology with human gene), and determined the cell proliferation, migration, and apoptosis. Results displayed that siRNA cocktail could silence the VEGF and HER2 better than single siRNA simultaneously, and the inhibition of cell proliferation, migration, and inducing apoptosis were good effects by the treatment of siRNA cocktail. Concurrently, the anti-angiogenesis had been evaluated on the HUVECs angiogenesis model; result showed that HUVECs were inhibited to form capillary tube-like structures on Matrigels as compared with the untreated group after siRNAs transfected (Fig. 7).

HER-2 could impact angiogenesis, and overexpression of HER2 is correlated with up-regulation of VEGF in breast cancer [34]. In the study, we also found that the HER2 inhibition could induce down-regulation of VEGF, but it does not vice versa in the gastric cancer cell, as shown in Fig. 1. In summary, the use of siRNA cocktail targeting VEGF and HER2 deserves further investigation as a novel approach to gastric cancer therapy.
